# Sterically Induced Enhancement in the Electrochemical Stability of Salen-Type Cathode Materials

**DOI:** 10.3390/polym17020178

**Published:** 2025-01-13

**Authors:** Julia V. Novoselova, Evgenii V. Beletskii, Daniil A. Lukyanov, Sofia S. Filippova, Uliana M. Rodionova, Vladimir V. Sizov, Elena V. Alekseeva, Oleg V. Levin

**Affiliations:** 1 Department of Chemistry, St. Petersburg University, Universitetskaya nab., 7/9, Saint Petersburg 199034, Russia; 2 Laboratory of Technologies of Materials and Devices of Electrochemical Energy Sources, Federal Research Center for Chemical Physics and Medicinal Chemistry, Russian Academy of Sciences, Avenue ac. Semenova, 1, Chernogolovka 142432, Russia

**Keywords:** nickel–salen polymers, supercapacitors, electrochemical stability, steric hindrance, water-induced degradation, cyclic voltammetry, operando conductance, impedance spectroscopy, X-ray photoelectron spectroscopy (XPS), density functional theory (DFT)

## Abstract

This study investigates the electrochemical degradation mechanisms of nickel–salen (NiSalen) polymers, with a focus on improving the material’s stability in supercapacitor applications. We analyzed the effects of steric hindrance near the nickel center by incorporating different bulky substituents into NiSalen complexes, aiming to mitigate water-induced degradation. Electrochemical performance was assessed using cyclic voltammetry, operando conductance, and impedance measurements, while X-ray photoelectron spectroscopy (XPS) provided insights into molecular degradation pathways. The results revealed that increased steric hindrance from methyl groups significantly reduced the degradation rate, particularly in water-containing electrolytes, by hindering water coordination to the Ni center. Among the studied polymers, the highly substituted poly[Ni(Saltmen)] exhibited superior stability with minimal capacity loss. Density functional theory (DFT) calculations further supported that steric protection around the Ni atom effectively lowers the probability of water coordination. These findings suggest that sterically enhanced NiSalen polymers may offer a promising path toward durable supercapacitor electrodes, highlighting the route of molecular engineering to enhance material stability.

## 1. Introduction

Electrochemical capacitors (ECs), or supercapacitors, constitute—along with batteries—the key technology for a fast-growing market of electric vehicles and portable electronic devices. Supercapacitors generate high specific power due to the high rate of charge and discharge, have a cycling life of up to 10^6^ cycles due to good reversibility, and demonstrate a relatively low temperature coefficient [1,2,3]. Yet, the specific energy stored in commercial supercapacitors is still inferior to that of batteries. The specific energy can be increased by either the development of electrode materials with a higher specific capacitance or by an increase in the operational voltage window by the proper selection of the electrolyte and the active material [4,5]. Both approaches can be implemented with conductive polymers, which often demonstrate high electrical conductance [6,7], specific capacitance [8], and a wide electrochemical activity window [9,10]. Along with the appealing mechanical properties of polymers [11], this enables the creation of flexible [12,13] and stretchable [14,15] supercapacitors as an additional benefit.

The prospective materials for supercapacitor electrodes reported to date include conducting polymers with extended systems of π–bonds, like polypyrrole [16,17], polyaniline [18,19], and polythiophene [20,21]. The performance of the electrodes based on conducting polymers can be enhanced by combining them with carbon nanotubes (CNTs) [22], graphene [23,24], metal oxides [25], and other inorganics [10,26] into a composite material. An alternative approach to performance enhancement implies endowing molecular ordering at a hundred-nanometer level by fabricating nanostructured materials in the form of nanoparticles [21,27] or nanowires [19]. An interesting class of prospective cathode materials in terms of molecular engineering is presented by polymerized transition metal complexes with salen-type ligands [28,29,30,31,32] (salen = N, N’-ethylene-bis (salicylidenimine)). These compounds can be considered an asymptotic case of combining a conductive polymer with an inorganic additive into a composite material. While the organic ligand secures the capacitive function of the material through a quinone-like redox chemistry, the central metal atom ensures conductance through d-π orbital interaction and affords nucleation centers for interlayer self-arrangement through d-d orbital interactions. In terms of applications as supercapacitor, polymerized nickel–salen complexes demonstrate high specific capacitance (200–300 F/g), a fast charge–discharge rate, and a reversible redox response of high stability in nonaqueous electrolytes with a wide electrochemical window, which ensures a large amount of energy stored by these materials [33,34].

Despite numerous examples of the application of [Ni(salen)] complexes in supercapacitors [24,29,35,36], reports on the cyclability of these materials are controversial. Data on the rate of electroactive response decay reported by different groups may differ by several orders of magnitude. Though all reported capacitors were based on nonaqueous electrolytes, any solvent inevitably contains water (at least in trace amounts), which, in turn, leads to a gradual degradation of the electrode material of poly-salen type and reduces the lifetime of the device [37,38]. The importance of water in the degradation of NiSalen polymers was highlighted on a concept level in our previous works [39,40,41] and has been widely discussed by other authors [42,43,44,45,46], but there is no common opinion on the mechanism of electroactivity loss. The hypotheses can be classified into “reactivity” and “transport” models of electroactivity decay in poly-salens. The “reactivity” hypotheses see the reason for the instability of the complex in the coordination of water by a central metal atom [42] or the stabilization or destabilization of the complex by the electronic effects of the substituents in the salen ligands [31,39,41], while “transport” models suggest the influence of polymer film thickness [46], doping and dedoping enhancement by sterically induced nanoporosity of the film [44], and water coordination-associated changes in the charge transfer parameters of poly-salen films [40]. Based on the proposed mechanism, one may assume that steric hindrance near the metal center may help to protect NiSalen polymers against the attack of nucleophiles, and, in particular, water.

In the present work, we aim to study the electrochemical activity, charge transfer parameters, and electronic conductance of NiSalen polymers with different structures in nonaqueous and water-containing electrolytes. We have performed systematic variation of the steric hindrance of the Ni atom by using NiSalen complexes with different positions and degrees of bulky substituents to reveal how steric hindrance affects the susceptibility of the complex to water. We have employed a set of methods including CV, operando conductance, and impedance measurements, as well as XPS analysis, to determine the pathways and rate of the electrochemical degradation of NiSalen-type polymeric films. Our results will provide a deeper understanding of the degradation processes in these polymers and help to improve the practical applicability of functional materials based thereon.

## 2. Materials and Methods

Monomeric complexes were synthesized and purified as described previously [47]. An electrolyte, 0.1 M tetraethylammonium tetrafluoroborate N(Et)_4_BF_4_ in anhydrous CH_3_CN (Sigma-Aldrich, St. Louis, MA, USA), was stored in an Ar-filled dry box over 3 Å activated molecular sieves for at least 10 days and filtered through a 0.22 µm membrane prior to use for “dry” experiments. By this pretreatment, water content was reduced to ca. 3 ppm of water according to Karl Fischer titration. For the experiments denoted as “wet”, 1% of deionized water was added prior to use. Cyclic voltammetry and electrochemical impedance spectroscopy (EIS) were carried out on an Autolab PGSTAT30 (Eco Chemie, Utrecht, The Netherlands) and Elins P-30I (Moscow, Russia) in sealed three-electrode cells filled with Ar, using a platinum plate with an area of 1 cm^2^ as an auxiliary electrode and BASi MF-2062 Ag in 0.1 M AgNO_3_ in CH_3_CN as a reference electrode. The potential of this electrode was calibrated as +0.4 V vs. Ag/AgCl. Experimental data of EIS were fitted by NOVA 1.11 Autolab software. Conductance measurements were performed using DropSens µSTAT 400 bipotentiostat (Metrohm, Oviedo, Spain) on interdigitated platinum electrodes G-IDEPT5 (Metrohm, Spain). X-ray photoelectron spectroscopy (XPS) was carried out on a Thermo Fischer Scientific (Waltham, MA, USA) Escalab 250Xi Spectrometer with nonmonochromatic AlKα radiation.

The polymeric films for CV and EIS experiments were synthesized on a 0.07 cm^2^ glassy carbon electrode from the 1 mM solution of monomeric complexes in electrolyte by potentiostatic oxidation at 0.8 V until the passage of charge equal to 0.95 C cm^−2^. Electrochemical studies were carried out using the cyclic voltammetry (CV) method at a range from −0.2 to 0.8 V with the potential sweep rate of 50 mV·s^−1^. For dry experiments, the water content after the completion of the experiment was determined by Karl Fischer titration. For XPS measurements, polymer films were deposited on a Pt working electrode S = 0.07 cm^2^ cycled at a range of potential from 0.2 V to 1.2 V in dry electrolyte for 5 cycles and then 50 cycles with the addition of 1% water. EIS measurements were performed using a frequency range from 0.01 Hz to 100 kHz. Initially, the impedance spectra were recorded in dry electrolyte (0.1 M Et_4_NBF_4_ in CH_3_CN) to establish a baseline. Then, 1% water was added to the electrolyte, and the polymer films were cycled four times to assess the effect of water on electrochemical performance.

For conductance measurements, the polymers were potentiostatically deposited on IDE electrodes at 0.8 V until the charge pass reached 20 mC. The stability tests combined with *operando* conductance measurement were carried out in CV mode at a range from −0.1 to 0.8 V with a scan rate of 5 mV·s^−1^ for 5 cycles in dry electrolyte and then for 10 cycles after the addition of 1% water. A potential gap of 10 mV was set between IDE combs to determine the conductance.

Direct electronic conductance measurements were performed on interdigitated electrodes simultaneously with cyclic voltammetry. An IDE electrode with a surface area of 0.34 cm^2^ was used, with the following geometry: single track length 6.76 mm, single track width 5 µm, array band gap 5 µm, number of tracks 250 for each working electrode. Cyclic voltammetry at 5 mV·s^−1^ was performed simultaneously on the IDE grids as two working electrodes with a constant difference of 10 mV between them. Currents passing through the working electrodes included the Faraday currents of the electrochemical process and the leakage Ohmic current.

To assess the polymer film conductance, it was assumed that the magnitude of the Faradaic currents is the same on both combs, so the leakage current between the combs was calculated according to the formulas [48]

I_WE №1_ = I_F_ − I, (1)

I_WE №2_ = I_F_ + I, (2)

(3) $$I=\frac{I_{WE\;№2}-I_{WE\;№1}}{2},$$

where I_WE №1_ и I_WE №2_—currents passing through the first and second combs; I—the leakage current between the combs.

Finally, conductance G and resistance R could be calculated according to the formula

(4) $$G=\frac{1}{R}=\frac{I}{V},$$

where *V*—the potential difference between the working electrodes.

The assumption of equality of Faradaic currents on both IDE combs used in the above derivation is valid for a relatively high conductance of the film. If the leakage current is too low, using Equation (4), we will derive the pseudoresistance resulting from the difference in Faradaic currents at the two working electrodes. This pseudoresistance repeats the shape of the 1st derivative of the cyclic voltammogram and may even appear to have a negative value. In this case, we will consider the film conductance to be below the detection limit.

DFT calculations were performed using the Gaussian 16 package [49]. Full geometry optimizations were carried out for low-spin and high-spin oxidized monomeric complexes with or without water molecules coordinated to the axial positions at the nickel atom. Pople’s 6-311+G* all-electron basis set was used for all atoms in conjunction with the CAM-B3LYP long-range-corrected hybrid functional [50]. Media effects were taken into account using the conductor-like polarizable continuum model (C-PCM) with acetonitrile as the solvent [51,52].

## 3. Results

**Figure 1:**
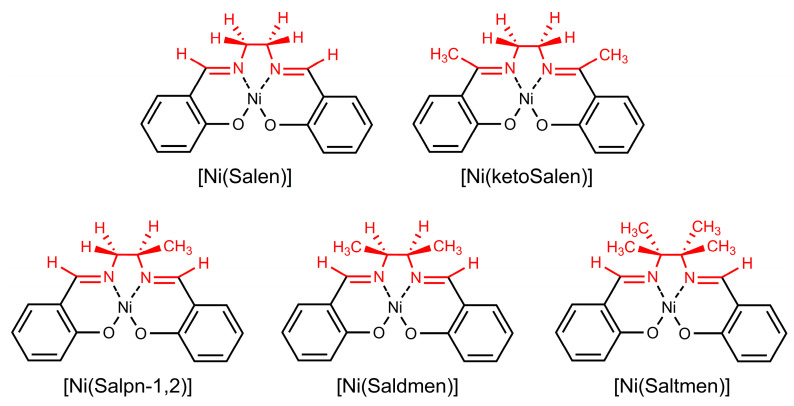
Structures and abbreviations of the monomers used for the preparation of the polymer-modified electrodes. Imine bridge fragments are colored in red.

To assess the impact of the imine fragment substitution on the stability of the polymeric NiSalens, five complexes were polymerized electrochemically, affording thin films of the corresponding polymers (Figure 1).

**Figure 2:**
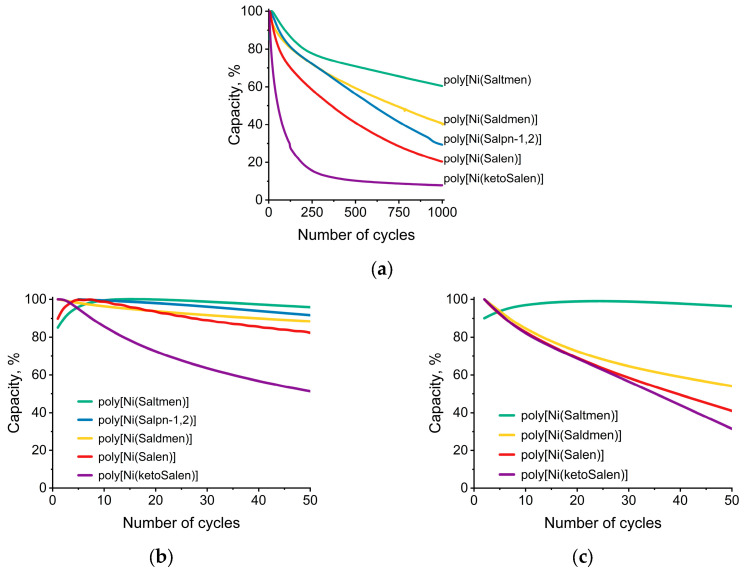
Relative capacity of electrodes modified by the polymeric complexes poly[Ni(Salen)], poly[Ni(Saltmen)], poly[Ni(ketoSalen)], poly[Ni(Salpn-1,2)], and poly[Ni(Saldme)] as functions of cycle number during charge–discharge in dried electrolyte 0.1 M N(Et)_4_BF_4_ in CH_3_CN: (**a**) 1000 cycles; (**b**) first 50 cycles; (**c**) wet electrolyte containing 1 wt% of water (voltage range from −0.2 V to 0.8 V vs. Ag|Ag^+^, AN).

The cycling stability of the films was estimated using relative capacity. This value indicates the reversible fraction of the charge passed through the system in a certain cycle. The long-term stability experiments in dried electrolyte showed that the increasing number of methyl groups in the bridge stabilizes the film, while an attachment of the methyl groups to the imine functional group leads to a dramatic decrease in stability (Figure 2).

**Figure 3:**
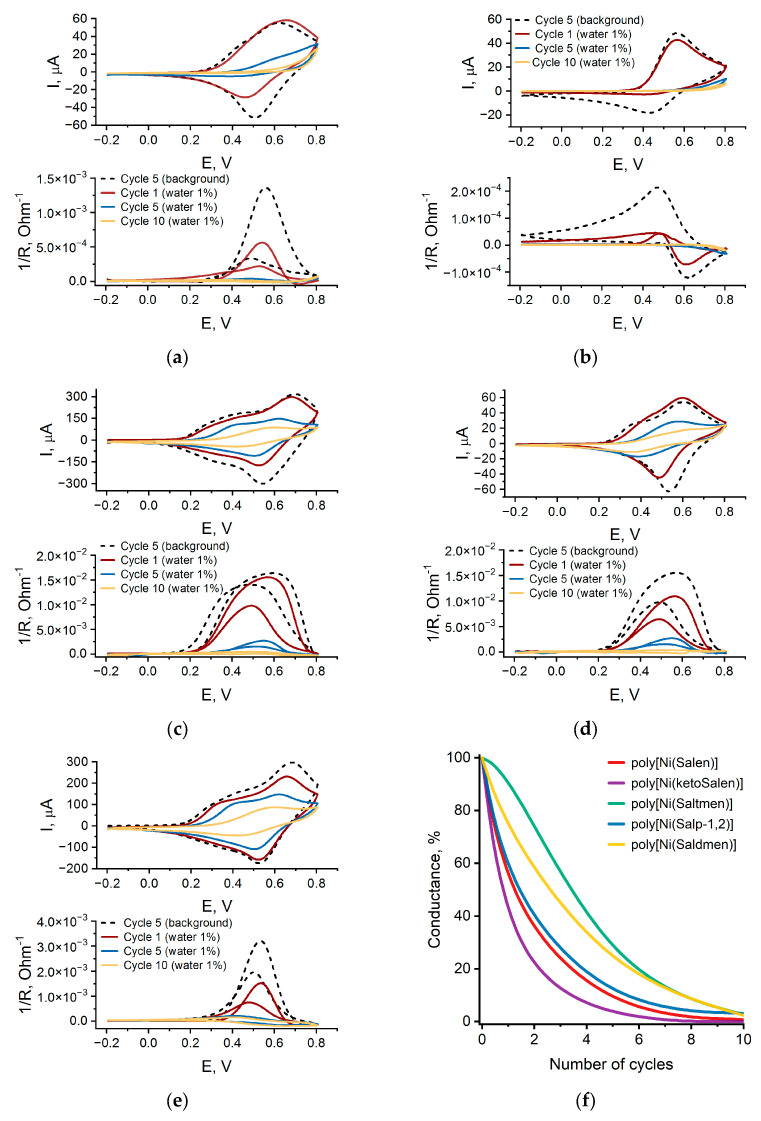
Cyclic voltammograms of (**a**) poly[Ni(Salen)], (**b**) poly[Ni(ketoSalen)], (**c**) poly[Ni(Saldmen)], (**d**) poly[Ni(Saltmen)], and (**e**) poly[Ni(Salpn-1,2)] in (Et_4_N)BF_4_ in CH_3_CN solution at 5 mV·s^−1^ and electronic conductance of the films measured in situ during CV recording; (**f**) maximum conductance on oxidation sweep vs. cycle number.

Initially, all materials showed close capacity values, which indicate the independence of normal charge–discharge processes on the bridge structure. In contrast, the conductance of the NiSalen polymers in dry electrolyte depends on the bridge structure: it improves with an increasing degree of the bridge substitution, while the substitution of the imine carbon leads to a decrease in conductance (Figure 3).

According to CV (Figure 2 and Figure 3), all materials show a gradual degradation of electrochemical capacity C and electrical conductance G in wet electrolyte from cycle to cycle. The highest degradation rate of both C and G is observed for poly[Ni(ketoSalen)], derived from the ketone imine ligand. For the bridge-methylated polymeric complexes, the C and G degradation rates decrease with an increasing number of -CH_3_ substituents. The most stable polymeric complex, poly[Ni(Saltmen)], shows literally no degradation of capacity but the compatible rate of conductance decreases.

The conductance of all polymers decreases much faster than capacity in wet electrolyte: while capacity retains at least 30% of the initial value after 50 CV cycles and does not reach a plateau, conductance falls to a plateau after 10 CV cycles, which means that conductance degrades faster than capacity. As described previously, such degradation behavior witnesses the interruption of the charge transport pathways along the polymer chains owing to the breaking of conjugation at particular chain units [40].

Films of NiSalen polymers are known to demonstrate high intrinsic electronic conductance [48] and counter ion diffusion coefficient in anhydrous electrolytes [28]. Electroactivity loss should be connected with changes in these charge transfer parameters. As was shown from the IDE measurements, electronic conductance is strongly affected by the presence of water.

**Figure 4:**
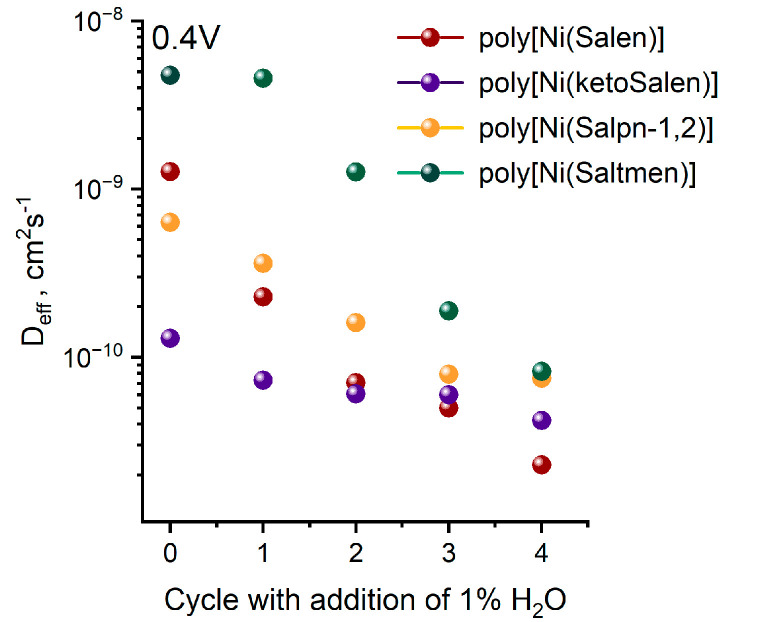
Binary diffusion coefficients estimated by Mathias–Haas model from EIS data at 0.4 V, (Et_4_N)BF_4_ in CH_3_CN.

To provide further insight into the charge transport process inside the polymer films and separate ionic and electronic contributions of charge transport to the overall conductance degradation, electrochemical impedance spectroscopy was utilized. The binary diffusion coefficient D_eff_ was calculated from EIS data using the Matthias–Haas model (Figure 4) [53].

**Figure 5:**
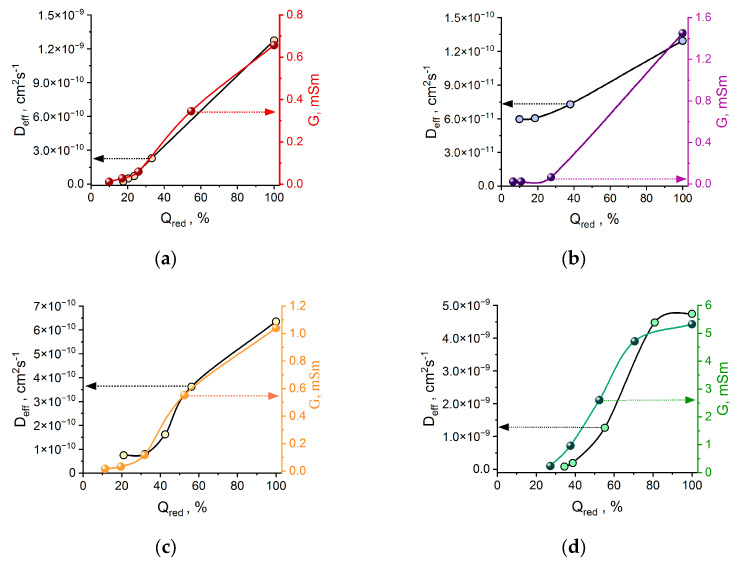
(**a**) Poly[Ni(Salen)]; (**b**) poly[Ni(ketoSalen)]; (**c**) poly[Ni(Salpn-1,2)]; (**d**) poly[Ni(Saltmen)].

In contrast to electrical conductance G, the binary diffusion coefficient D_eff_ depends on both the motion of electrons and charge-compensating ions in the film and thus characterizes an overall charge motion in the film swelled with electrolyte. To make a direct comparison of the G and D_eff_ values, these values were brought to a common scale of the residual charge of the polymer film after each cycle because it reflects the cumulative changes in the film’s properties (Figure 5). In the series of bridge-substituted NiSalen polymers, the degradation of D_eff_ correlates with the degradation of G, which means that the loss of electrical conductance is the main factor limiting charge transport degradation. In the case of poly[Ni(ketoSalen)], the D_eff_ value remains nearly the same while G degrades rapidly, indicating that the charge motion in this polymer is limited by ion transport.

The observed behavior of NiSalen polymers with different degrees of steric hindrance additionally supports our previously reported mechanism [40], where the Ni atom is the site for the H_2_O attack. Indeed, the electrical conductance of the polymeric complexes decreases faster than their Faradaic capacity, which indicates the breakdown of the charge transport pathways in the polymer chains rather than the bulk degradation of the electroactivity of the polymer.

To assess the macroscopic hydrophobicity of the polymers and its possible correlation with water-promoted degradation, we measured the contact angles of wetting with H_2_O using the static sessile drop method (Table S1). The contact angle for poly[Ni(Salen)] was 35°, indicating very good wetting of the film. The contact angles decreases in the order of poly[Ni(Salen)]–poly[Ni(Salpn-1,2)]–poly[Ni(Saldmen)] (35°–31°–28°), which indicates that the addition of one or two methyl groups does not increase the hydrophobicity of the film. The contact angle of poly[Ni(ketoSalen)] has an intermediate angle value of 33°. However, poly[Ni(Saltmen)], with four methyl groups, appears to be much more hydrophobic, with a contact angle as high as 63°. Summarizing this, we observe no direct correlation of the hydrophobicity of the polymer films with the bulkiness of their imine bridge until some threshold, where hydrophobicity is suddenly increased for fully substituted bridge. It justifies the drastic increase of stability of poly[Ni(Saltmen)] in water-containing electrolytes compared to other studied materials.

### 3.1. XPS

**Figure 6:**
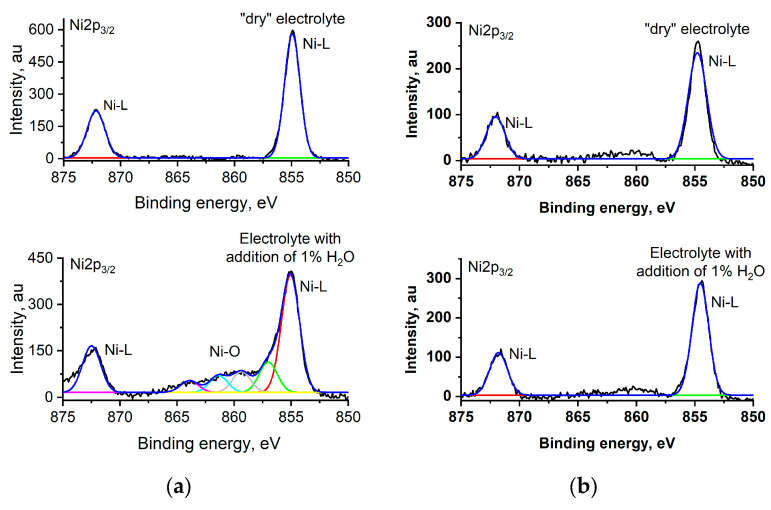
Ni2p_3/2_ XPS spectra of polymer films cycled in water-free and water-containing electrolytes: (**a**) poly[Ni(Salen)]; (**b**) poly[Ni(Saltmen)].

The coordination of water to the Ni metal center should be marked on the surface of polymers and can be detected by XPS technique. We performed XPS analysis of the surface of the most representative polymers, poly[Ni(Salen)] and poly[Ni(Saltmen)]. Of the greatest interest are the nickel Ni2p_3/2_ spectra, since they provide information concerning coordination to the metal center (Figure 6).

The Ni 2p_3/2_ spectrum of the studied complexes cycled in dried electrolyte contains two characteristic peaks, typical for nickel–salen complexes (872.7 eV and 855.6 eV), which are assigned to Ni(II) 2p_1/2_ and Ni 2p_3/2_ electrons in square-planar coordination compounds [54,55]. After cycling in water-containing electrolyte, the Ni spectrum changes, depending on the structure of the complex. For poly[Ni(Salen)], new peaks appear in the region from 856 eV to 865 eV. These peaks, corresponding to Ni–O bond in an octahedral coordination environment, are similar to nickel hydroxide peaks [56,57,58,59]. The appearance of these peaks indicates the axial coordination of water or some oxygen-containing species to the Ni atom after treatment in water-containing electrolyte. This transforms the Ni complex from square-planar to octahedral, breaking the interchain conjugation pathways and thus leading to a decrease in polymer conductance. On the contrary, in the case of the most stable among the studied polymers, poly[Ni(Saltmen)], Ni spectrums after cycling in «dry» and water-containing electrolytes are similar, indicating that the square-planar configuration of the complex remains intact.

**Figure 7:**
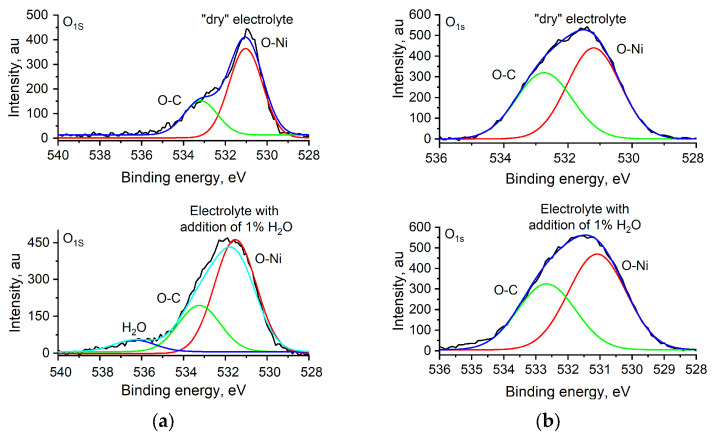
O1s XPS spectra of polymer films cycled in water-free and water-containing electrolytes: (**a**) poly[Ni(Salen)]; (**b**) poly[Ni(Saltmen)].

To confirm this assumption, we analyze the O_1s_ spectrum of poly[Ni(Salen)] and poly[Ni(Saltmen)] films (Figure 7).

**Figure 8:**
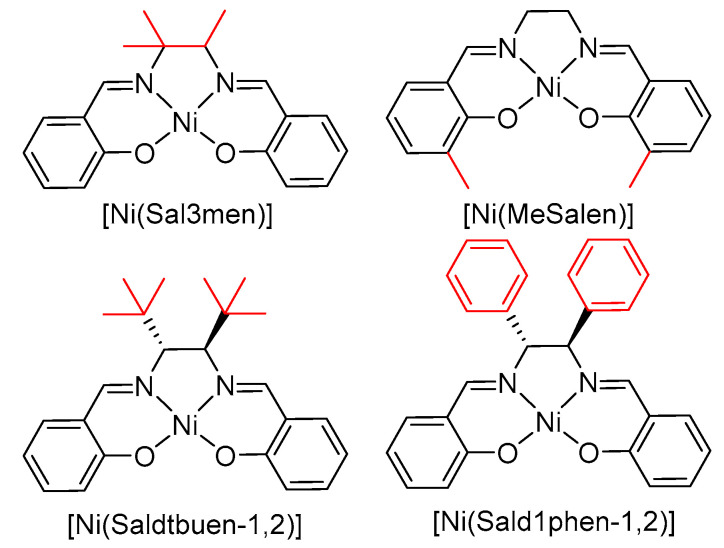
Computationally studied complexes of NiSalen series. Imine bridge fragments are colored in red.

The XPS O_1s_ spectra of poly[Ni(Salen)] and poly[Ni(Saltmen)] films recorded after cycling in «dry» electrolyte are similar. The spectra contain two peaks at 531 eV and 533 eV. The first peak is assigned to an O-Ni bond in the Ni–salen complex [55,60]. The second peak corresponds to the O–C bond [61] from a Schiff base ligand. The second peak corresponds to the O–C bond [61] from a Schiff base ligand. After cycling in water-containing electrolyte, the peak at 531 eV in the poly[Ni(Salen)] O1s spectrum remains almost the same. But the shoulder of the second peak at 533 eV becomes smoother, which corresponds to a decrease in C–O bond weight, which, in this case, may be attributed to the increase in the number of oxygen-containing ligands. Moreover, a new peak at 536.4 eV appears on the O_1s_ spectrum. The peak may be assigned to water coordinated to the Ni center of the poly[Ni(Salen)] complex [62]. In the case of poly[Ni(Saltmen)], additional oxygen coordination on nickel during cycling in water-containing electrolyte was not detected, as the O_1s_ spectrum is similar to the spectrum recorded after cycling in a water-free electrolyte. The peak may be assigned to water coordinated to the Ni center of the poly[Ni(Salen)] complex [62] (Figure 8).

As a result, XPS data support previous assumptions that, during cycling in a water-containing electrolyte, water is coordinated on the nickel atom as an axial ligand. In the case of poly[Ni(Salen)] and poly[Ni(ketoSalen)], this process is not inhibited by any steric effect, and new intensive peaks of the Ni–O bond appear. The poly[Ni(Salpn-1,2)] complex has one methyl group in the diimine bridge, located outside of the complex plane, and it makes water coordination less effective, so the new peaks of Ni-O are less intensive. The four methyl groups in the bridge of the poly[Ni(Saltmen)] complex sterically protect nickel (Ni) from the coordination of water, so XPS spectra of the polymer remain intact after treatment in a water-containing solution.

### 3.2. DFT Calculations

Geometry optimizations of mononuclear one-electron-oxidized complexes with two coordinated water molecules confirm the possibility of H_2_O coordination to the axial positions at the nickel atom. For all complexes, the coordination of water was accompanied by a significant negative energy change. However, the calculated energies do not demonstrate a straightforward correlation with the experimental data on the stability of the complexes considered in the present study. In fact, the energy change upon water coordination is largely similar for most of the complexes, namely, [Ni(Salen)] (−63 kJ/mol), [Ni(ketoSalen)] (−58 kJ/mol), [Ni(Salpn-1,2)] (−61 kJ/mol), [Ni(SaldMe)] (−57 kJ/mol), and [Ni(Saltmen)] (−52 kJ/mol). It is worth noting that the stepwise introduction of methyl substituents into the ethylene-bridging fragment of the original salen ligand leads to an increasingly less favorable energy change accompanying the coordination of water molecules to the metal site: +2 kJ/mol relative to [Ni(Salen)] for [Ni(Salpn-1,2)], +6 kJ/mol for [Ni(SaldMe)], and +11 kJ/mol for [Ni(Saltmen)]. However, only the energy difference for [Ni(Saltmen)] appears to be greater than the appreciated computational accuracy of the method.

The stability of low-spin and high-spin forms of the complexes with axial water molecules is very similar, with a typical energy difference of 3 kJ/mol or less, which suggests that both forms can potentially coexist in comparable quantities. Interestingly, the differences in electronic structure between low-spin and high-spin species are mostly limited to the salen-type ligand, while the total charge and spin density on the nickel atom and coordinated water molecules are essentially the same for both forms.

The absence of a well-defined correlation between the experimental electroactivity data and computed energy effects of water coordination suggests that the stability of the complexes is determined by non-thermodynamic factors. In other words, if water molecules reach the metal site, the formation of an aquacomplex is likely to occur with high probability, which does not show a strong dependence on the composition of the complex. Therefore, the accessibility of the metal site emerges as the most likely factor controlling the stability of the complexes in solutions containing trace amounts of water. The abovementioned decrease in energy change upon water coordination to [Ni(Saltmen)], for which the attachment of axial H_2_O ligands is the least favorable of all complexes, provides indirect support for the latter statement. Unlike other complexes with fewer methyl substituents in the bridging fragment, in [Ni(Saltmen)], the steric loading of the bridge finally becomes sufficiently high to significantly affect the coordination of water to the metal center, i.e., the accessibility of nickel is hindered even locally, on the intramolecular level. For [Ni(Salpn-1,2)] and [Ni(SaldMe)], this effect is less pronounced, but for either of these two complexes, the coordination of water is also less favorable than for [Ni(Salen)], which is likely to be caused by the same steric factor.

To illustrate the key factor influencing water binding to the said complexes, we excerpted some indicative properties related to electronic and steric effects in the complexes described in the manuscript. In addition to that, similar data are also provided for a number of related compounds, which were considered only computationally and therefore were not mentioned in the text (Figure 8).

The electronic effects of the substituents introduced into the structure of salen-type nickel complexes can be considered effects on electron density over the molecule. Talking about coordination to the Ni atom, we describe the electronic effects (in a comparative sense) through the comparison of effective charge on the Ni atom in oxidized species. The charge on Ni was obtained by applying the Natural Population Analysis procedure to the computational data produced using the methods described in the manuscript (Table 1). One can easily observe the subtle effect of the bridge substituents on the computed charge: the largest change relative to [NiSalen]^+^ is found for [NiSatmlen]^+^, and it is equal to only 0.012 e (or 1.5%). Moreover, the computed charge becomes more positive upon the addition of substituents to the bridging fragment. As a result, the electronic effects seem to work opposingly to the experimental observations.

**Figure 9:**
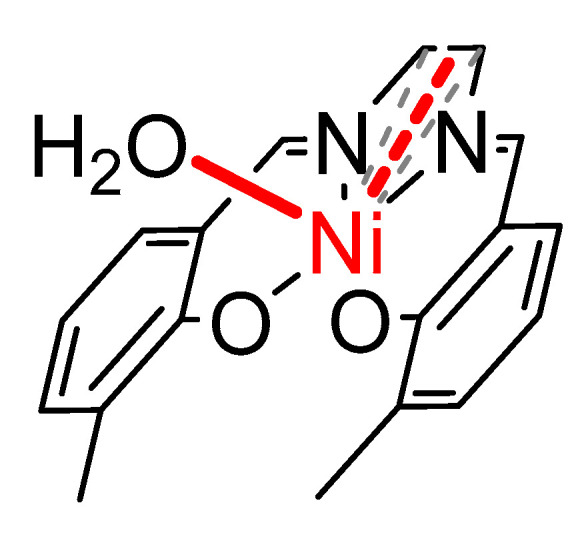
Determination of ∠ X-Ni-O (red angle).

The steric effects present a serious challenge for quantitative computational evaluation, so here, we use a more intuitive geometrical criterion, namely, the angle between the Ni-OH_2_ bond and the median of the C(bridge)-Ni-C(bridge) angle (∠ X-Ni-O, Table 1, Figure 9. The angle provided in the table is the averaged value for two coordinated water molecules from both sides of the complex plane. The values of this angle, which are greater than 90°, indicate a tilt of the axial ligand away from the bridging fragment. The second property, which can also be related to steric effects, is the “O-H distance”, namely averaged distance from the water oxygen to the nearest hydrogen atom of the bridge. One can see from the table that the X-Ni-O angle tends to increase with increasing number of methyl groups in the bridging fragment. For complexes with bulky substituents, such as phenyl or *t*-butyl groups, this angle may exceed 100 degrees. At the same time, the O-H distance decreases with increasing steric loading from the bridge, and for tri-, tetramethyl-, and *t*-butyl-bridged complexes, it is lower than the sum of Van der Waals radii of O and H, providing a strong indication of a sterically driven mechanism.

**Figure 10:**
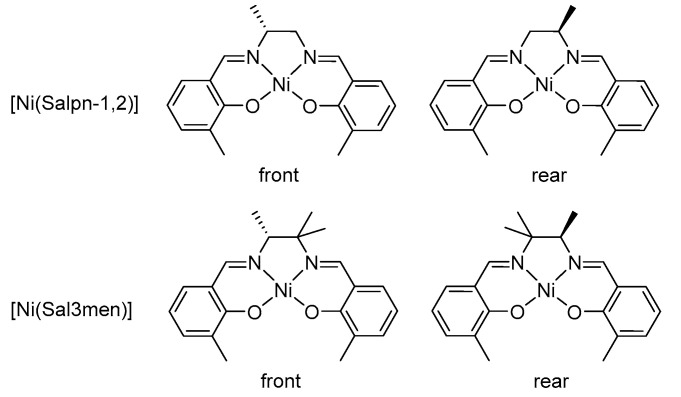
Front and rear sides of the [Ni(Salpn-1,2)] and [Ni(Sal3men)].

In addition, for the [Ni(Salpn-1,2)] and [Ni(Sal3men)] complexes with nonequal coordination sites due to the low symmetry of the complex (Figure 10, two H_2_O ligands show nonequal coordination geometry. Coordination to the less hindered “front” side (0 methyls in the case of [Ni(Salpn-1,2)] and 1 methyl in the case of [Ni(Sal3men)]) proceeds with lower ∠ X-Ni-O values and higher O-H distances, compared with coordination to the more hindered “rear” side (1 methyl in the case of [Ni(Salpn-1,2)] and 2 methyls in the case of [Ni(Sal3men)]) (Table 1). Differences in ∠ X-Ni-O and O-H distance amount to 7.9° and 0.228 Å in the case of [Ni(Salpn-1,2)] and 1.7° and 0.062 in the case of [Ni(Sal3men)]. This phenomenon cannot be explained by electronic effects and appears to be a strong argument for the steric factors.

Concluding this, the role of electronic effects appears to be relatively small and likely works oppositely to the experimentally observed water coordination trend. In contrast to this, the role of steric effects, which is estimated here via structural metrics obtained from DFT calculations, is in reasonable agreement with the experiment.

## 4. Discussion

In this work, it was determined that electronic conductance in NiSalen polymers is the main parameter that influences their electroactivity in water-containing electrolytes. We obtained additional evidence for the metal-centered degradation process of NiSalen polymers in the presence of water, originating from electrochemical and XPS data. Additionally, we observed that the introduction of one or two -CH_3_ groups results in only minor protection of the polymer, while four methyl groups protect it to a much higher extent. A possible explanation is that the introduction of up to two -CH_3_ groups out of four possible positions cannot cover the metal center with a continuous protection of the metal center, while four -CH_3_ groups cover both sides of the complex plane from both possible positions. The introduction of -CH_3_ substituents into the imine carbon atoms, on the contrary, increases their degradation rate during cycling in both dried and water-contaminated electrolyte. This may be explained by an increase in the total electron density of the complex, which facilitates the coordination of water to the metal center, while the steric hindrance caused by the substituents which lie in the plane of the complex is negligible. The provided explanation is supported by the DFT calculations of the coordination of water to the metal center.

We then demonstrated that the steric protection of the Ni atom by methyl substituents in the diamine bridge allow the degradation process to slow down and enable polymer operation even in water-containing electrolytes. The protection effect strongly depends on the number of methyl substituents in the diamine and works best with four -CH_3_ groups. However, steric protection can only decrease the degradation rate but never prevent it. The possible pathway for further improvement to the electrochemical stability of the NiSalen polymers against water-promoted degradation may be the combination of the previously described stabilization with three substituents at the benzene rings with the approach present in this work.

## Figures and Tables

**Table 1:** Excerpted computational results for the considered complexes.

	Natural Charge on Ni, e	∠ X-Ni-O, °	O-H Distance, Å
[Ni(Salen)]	0.790	94.4	3.168
[Ni(Salpn-1,2)] front	0.791	88.4	2.828
[Ni(Salpn-1,2)] rear	0.791	96.3	2.600
[Ni(Sadmlen)]	0.791	95.4	2.760
[Ni(Sal3men)] front	0.795	94.3	2.405
[Ni(Sal3men)] rear	0.795	96.0	2.467
[Ni(Satmlen)]	0.802	96.9	2.469
[Ni(KetoSalen)]	0.789	94.2	3.153
[Ni(MeSalen)]	0.773	92.1	3.048
[Ni(Saldtbuen-1,2)]	0.799	102.2	2.225
[Ni(Sald1phen-1,2)]	0.786	97.7	2.666

## Data Availability

The original contributions presented in this study are included in the article. Further inquiries can be directed to the corresponding author.

## Supplementary Materials

The following supporting information can be downloaded at https://www.mdpi.com/article/10.3390/polym17020178/s1: Table S1: Contact angles of wetting polymers with H_2_O.
