# Supplementary material for: Sterically Induced Enhancement in the Electrochemical Stability of Salen-Type Cathode Materials
Source: Polymers (Basel). 2025 Jan 13;17(2):178. doi: 10.3390/polym17020178 (PMC11769176; doi:10.3390/polym17020178)
Supplement: Supplementary file 1 [file polymers-17-00178-s001.zip › polymers-3318355-supplementary.pdf]

Supporting Information

Sterically induced enhancement in electrochemical stability of salen type cathode materials

Julia V. Novoselova [1], Evgenii V. Beletskii [2], Daniil A. Lukyanov [1], Sofya S. Filippova[1], Uliana M. Rodionova[1], Vladimir V. Sizov [1], Elena V. Alekseeva [1,*], Oleg V. Levin [1]

Table S1. Contact angles of wetting polymers with H_2O.		
poly[Ni(Salen)]	poly[Ni(ketoSalen)]	poly[Ni(Salpn-1,2)]
35°	33°	31°
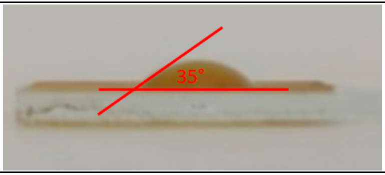	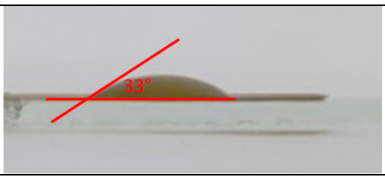	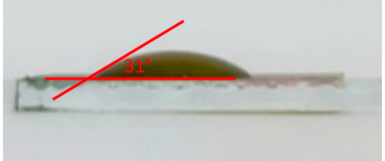
poly[Ni(Saldmen)]	poly[Ni(Saltmen)]	
28°	63°	
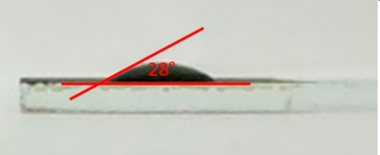	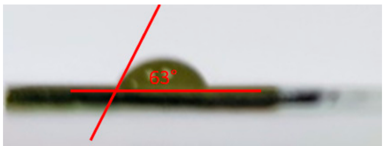	
